# Editorial: The pharmacological effects and mechanisms of drugs against human diseases by modulating redox homeostasis, volume III

**DOI:** 10.3389/fphar.2026.1868202

**Published:** 2026-05-12

**Authors:** Xiaofang Zhang, Xiao Cui, Ming Xu

**Affiliations:** 1 Academician Collaborative Laboratory for Basic Research and Translation of Chronic Diseases, Central Laboratories, The First Affiliated Hospital of Jinan University, Jinan University, Guangzhou, China; 2 Guangdong Provincial Key Laboratory for Chronic Disease Interactive Network Research, Jinan University, Guangzhou, China; 3 The 2nd Affiliated Hospital & Yuying Children’s Hospital of Wenzhou Medical University, Wenzhou, China; 4 School of Data Science, Shimonoseki City University, Shimonoseki, Japan

**Keywords:** disease, drugs, homeostasis, oxidatie stress, reactive oxygen species (ROS)

## Introduction

Redox homeostasis is a fundamental determinant of cellular and organismal health. Reactive oxygen species (ROS), reactive nitrogen species (RNS), glutathione-dependent buffering systems, thioredoxin networks, mitochondrial respiration, and redox-sensitive signaling proteins collectively regulate proliferation, metabolism, immunity, differentiation, and tissue repair. Under physiological conditions, controlled oxidant flux functions as an essential signaling modality regulating proliferation, immunity, and adaptation, whereas excessive ROS promotes cellular injury (Schieber et al., 2014). However, when oxidant production exceeds buffering capacity, or when antioxidant defenses are compromised, oxidative imbalance can drive lipid peroxidation, DNA injury, protein misfolding, mitochondrial dysfunction, chronic inflammation, and progressive tissue remodeling. Such mechanisms contribute broadly to cardiovascular disease, metabolic disorders, inflammatory syndromes, neurodegeneration, cancer, and aging (Sies et al., 2017; Sies, 2015; Forman and Zhang, 2021).

The Research Topic *“The pharmacological effects and mechanisms of drugs against human diseases by modulating redox homeostasis, Volume III”* captures the evolution of this field from conventional antioxidant theory toward mechanism-based redox pharmacology. The five studies included in this volume collectively demonstrate that successful therapies rarely function by indiscriminate radical scavenging alone. Instead, benefit is achieved through coordinated regulation of metabolism, mitochondrial function, endogenous antioxidant systems, immune responses, and inflammatory signaling pathways (Zhang et al.; Bigagli et al.; Qian and Wang; Nandagopal and Manickam; Shantanu et al.).

## Redox homeostasis as a therapeutic systems framework

To integrate the findings of this Research Topic, we propose a conceptual model termed “the Redox Homeostasis Therapeutic Matrix”, composed of four interacting domains.

First, pathological oxidant sources include mitochondrial electron leakage, NADPH oxidases, xanthine oxidase, cytochrome P450 enzymes, inflammatory leukocytes, and dysregulated nutrient metabolism.

Second, endogenous resilience systems comprise glutathione biosynthesis, methionine cycling, thioredoxin/peroxiredoxin pathways, NADPH regeneration, mitochondrial quality control, and adaptive transcriptional programs such as NRF2.

Third, downstream pathological outputs include NF-κB activation, inflammasome signaling, epithelial barrier dysfunction, fibrosis, apoptosis, ferroptosis, insulin resistance, and maladaptive immune polarization.

Fourth, pharmacological intervention nodes involve source-selective ROS suppression, restoration of antioxidant capacity, metabolic rewiring, immunoredox modulation, and organelle protection.

Each article in this Research Topic addresses one or more of these domains, underscoring the systems-level nature of redox medicine.

## Immunometabolic regulation in intestinal inflammation

A notable contribution by Zhang and colleagues employed time-resolved metabolomics in dextran sulfate sodium (DSS)-induced colitis to characterize metabolic remodeling across colon tissue, mesenteric lymph nodes, and serum during disease progression (Zhang et al.). The investigators identified progressive activation of purine metabolism, closely associated with escalating immune-inflammatory responses. They further recognized trigonelline as a circulating metabolite correlated with disease severity. Importantly, treatment with trigonelline or the purine metabolism inhibitor mycophenolic acid markedly alleviated histological injury, reduced inflammatory infiltration, and restored Th17/Treg balance (Zhang et al.).

This study is highly relevant to redox pharmacology because purine metabolism intersects with ATP turnover, uric acid generation, mitochondrial function, and inflammatory signaling. Excess purine catabolism can amplify oxidative stress, while altered nucleotide availability shapes immune-cell proliferation and differentiation. The observed correction of Th17/Treg disequilibrium further supports the concept that immune phenotypes are metabolically encoded. Rather than acting solely as anti-inflammatory agents, metabolic interventions may restore immune tolerance by normalizing redox-metabolic circuitry.

More broadly, this work highlights the value of multi-organ metabolomics for identifying actionable therapeutic pathways in inflammatory disease.

## Rebuilding endogenous antioxidant defenses in alcohol-related injury

Another important study by Nandagopal and Manickam investigated chronic ethanol-induced hepato-pancreatic injury and tested co-supplementation with S-adenosyl-L-methionine (SAMe) and B vitamins (Nandagopal and Manickam). Chronic ethanol exposure induced severe oxidative stress, impaired endogenous antioxidant defenses, increased inflammatory mediators, and caused significant structural injury in both liver and pancreas. Combined SAMe and B-vitamin treatment restored glutathione homeostasis, reduced inflammatory signaling, and markedly attenuated organ pathology (Nandagopal and Manickam). Because glutathione is the principal intracellular redox buffer, maintenance of its biosynthetic capacity is central to cellular stress resistance (Lu, 2013).

This study emphasizes a key lesson from modern redox biology: effective therapy often requires restoration of intrinsic antioxidant infrastructure rather than provision of exogenous antioxidants alone. SAMe is central to methylation reactions and transsulfuration pathways, whereas vitamins B6, B12, and folate support homocysteine metabolism and cysteine generation, thereby sustaining glutathione synthesis. Because glutathione is a principal intracellular redox buffer, replenishing its biosynthetic capacity can improve resilience across multiple disease states.

The implications extend beyond alcohol-associated disease to steatotic liver disease, pancreatitis, neurodegeneration, and cardiometabolic disorders characterized by one-carbon metabolic dysfunction and glutathione depletion.

## Source-targeted redox interventions

The third article in this volume (Qian and Wang) reinforces the importance of targeting upstream sources of pathological oxidants rather than relying exclusively on downstream scavenging. Disease-specific ROS generators such as NOX enzymes, uncoupled nitric oxide synthase, CYP isoforms, and dysfunctional mitochondria often sustain chronic tissue injury. Pharmacological suppression of these sources can reduce oxidative burden while preserving physiological redox signaling.

This principle has broad translational significance. For example, xanthine oxidase inhibition in hyperuricemia, NOX modulation in vascular disease, and CYP2E1 suppression in toxin-induced injury all exemplify source-selective redox therapy. By intervening closer to the origin of pathological oxidants, such strategies may offer greater efficacy and fewer unintended effects than non-selective antioxidants.

## Mitochondrial preservation and organelle crosstalk

The fourth contribution (Nandagopal and Manickam) highlights mitochondria as both generators and victims of oxidative stress. Mitochondrial dysfunction reduces ATP production while increasing electron leakage and ROS formation, thereby creating a self-reinforcing energetic-redox crisis. Oxidative injury may also impair endoplasmic reticulum function, calcium handling, lysosomal integrity, and autophagic flux.

Therapeutic preservation of mitochondrial quality—through enhancement of mitophagy, stabilization of membrane potential, improvement of substrate oxidation, or reduction of mitochondrial ROS—therefore represents a central strategy in redox medicine. Such approaches are relevant to ischemia-reperfusion injury, metabolic syndrome, heart failure, neurodegeneration, and chronic inflammatory diseases.

The inclusion of this theme in the present volume reflects the growing recognition that redox signaling is inseparable from organelle biology.

## Immunoredox modulation and resolution pharmacology

The fifth study (Nandagopal and Manickam) further underscores the intimate relationship between redox balance and immune regulation. ROS are critical for host defense, leukocyte migration, cytokine signaling, and pathogen killing. Yet excessive or persistent oxidant generation sustains macrophage activation, neutrophil-mediated tissue injury, T-cell dysregulation, and chronic inflammation.

Accordingly, drugs that recalibrate immune redox tone may promote resolution rather than simple suppression of inflammation. Examples include agents that shift macrophages toward reparative phenotypes, normalize neutrophil oxidative burst, restore regulatory T-cell function, or limit inflammasome activation. This emerging field of resolution pharmacology may be especially valuable in autoimmune, pulmonary, cardiovascular, and gastrointestinal disorders.

## Why many classical antioxidant trials failed

The studies in this Research Topic collectively help explain why conventional antioxidant supplementation frequently produced disappointing clinical results.

First, ROS are essential signaling molecules and should not be globally eliminated. Second, many antioxidants fail to accumulate in relevant intracellular compartments. Third, they do not address upstream ROS generators. Fourth, oxidative stress is usually intertwined with inflammation, metabolic dysfunction, and impaired repair pathways. Fifth, timing matters: transient ROS may be adaptive early in disease but pathological later.

In contrast, the five studies in this volume exemplify a new generation of strategies that target mechanisms rather than molecules.

## Toward precision redox medicine

The future of this discipline lies in patient stratification. Not all individuals display the same redox phenotype. Some may exhibit glutathione depletion, others mitochondrial ROS excess, ferroptotic lipid peroxidation, inflammatory NADPH oxidase activation, or reductive stress. Precision approaches should integrate biomarkers such as GSH/GSSG ratios, lipid peroxidation products, redox metabolomics signatures, mitochondrial DNA damage markers, and transcriptomic indices of NRF2 or inflammatory activation.

The metabolomics-guided approach of Zhang et al. is especially instructive, demonstrating how systems profiling can identify disease-stage-specific therapeutic nodes. Likewise, the metabolic rescue strategy of Nandagopal and Manickam (Nandagopal and Manickam) illustrates how biochemical phenotyping may guide nutrient-pharmacological combinations.

## Conclusion

The five studies assembled in *The pharmacological effects and mechanisms of drugs against human diseases by modulating redox homeostasis–Volume III* collectively show that redox homeostasis is not a narrow biochemical concept but a systems-level determinant of disease susceptibility and therapeutic response. Whether through modulation of purine metabolism and immune balance in colitis (Zhang et al.), restoration of glutathione biosynthesis in ethanol-induced hepato-pancreatic injury (Nandagopal and Manickam), source-selective oxidant suppression (Qian and Wang) mitochondrial preservation (Nandagopal and Manickam), or immunoredox recalibration (Shantanu et al.), these studies converge on a common principle: the most effective therapies restore dynamic redox equilibrium rather than merely suppress oxidation.

As metabolomics, spatial biology, and systems pharmacology continue to advance, redox medicine is poised to transition from empirical antioxidant use toward precision-guided, mechanism-based therapeutics. The contributions in this volume represent meaningful progress toward that future.
